# Tuneable multidirectional mechanical attributes of novel sectionally nonlinearly functionally graded femur and cranial bone implants with triply periodic minimal surfaces

**DOI:** 10.1371/journal.pone.0332104

**Published:** 2025-09-09

**Authors:** Nguyen Van Viet, Wael Zaki, Marwan El-Rich

**Affiliations:** 1 Mechanical and Nuclear Engineering Department, Khalifa University of Science and Technology, Abu Dhabi, United Arab Emirates; 2 Smart and Architected Materials Laboratory, Khalifa University of Science and Technology, Abu Dhabi, United Arab Emirates; IIIT Kurnool: Indian Institute of Information Technology Design and Manufacturing Kurnool, INDIA

## Abstract

Sectionally nonlinearly functionally graded (SNFG) structures with triply periodic minimal surface (TPMS) are considered ideal for bone implants because they closely replicate the hierarchical, anisotropic, and porous architecture of natural bone. The smooth gradient in material distribution allows for optimal load transfer, reduced stress shielding, and enhanced bone ingrowth, while TPMS provides high mechanical strength-to-weight ratio and interconnected porosity for vascularization and tissue integration. Wherein, The SNFG structure contains sections with thickness that varies nonlinearly along their length in different patterns. And TPMS scaffolds are smooth, porous structures that repeat in three dimensions and have zero mean curvature, offering high surface area and tuneable properties. This study presents a novel design and numerical analysis of SNFG titanium alloy Ti6Al4V femur and cranial bone implants incorporating TPMSs. The accuracy of the numerical model is validated through experiments and force-reaction analysis in terms of elastic stiffness of the white Polylactic Acid (PLA)-based SNFG femur and cranial bone implants, demonstrating good agreement among methods, having a maximum percentage difference of 15.6%. It is found that among various TPMS topologies, the gyroid structure is the most suitable candidate for manufacturing SNFG bone implants, offering superior multidirectional mechanical performance. Interestingly, the anisotropy and magnitude of elastic stiffness can be tailored to closely match natural bone by adjusting the gradient index and trabecular part length while maintaining a yield strength higher than that of bone. Additionally, during service, the implant may be subjected to an impact that generates mechanical waves propagating through its structure. These waves transmit the force impulse and induce the propagation of mechanical stress throughout the implant body. The result indicates that increasing the gradient index reduces shear and longitudinal stress wave velocities with minimal impact on wave velocity anisotropy, a key factor in enhancing implant longevity and performance. And, TPMS implants exhibit extreme multiaxial yield strength anisotropy, but it can be accurately captured using the extended Hill’s criterion, which provides a reliable and cost-efficient method for constructing the critical yield surface of SNFG femur and cranial titanium implants, helping to prevent permanent plastic deformation during service. Overall, this work lays the foundation for futuristic optimization approach aimed at designing ideal SNFG titanium femur and cranial bone implants with TPMSs for biomedical applications.

## Introduction

Nonlinearly functionally graded materials (FGMs) are advanced materials engineered with a gradual variation in composition and structure, optimizing their properties for specific applications, including biomedical engineering, energy absorption, impact resistance, and aerospace technology [1,2]. Dedicated attempts have been devoted to investigating the new properties of these materials, particularly in their integration with advanced porous architected topologies for innovative applications. In this context, Nian et al. [3] investigated the possibility of a functionally graded lattice-based composite materials, reporting that structure with graded lattice achieve more superior Pareto-optimal solutions than traditional structures. In the same attempt, Zhao et al. [4] numerically and experimentally examined the influence of gradient direction on the energy absorption and mechanical performance of FG sheet-typed structures. Their findings revealed that porosity gradations play a crucial role in biomedical applications that the structure’s thinnest side, with its highest surface area, enhances fluid flow and supports bone cell development for maximized nutrient delivery, meanwhile the thickest part offers maximum strength to shelter the implant. Furthermore, the thickness is able to be adjusted to make the implant’s stiffness close to the stiffness of the surrounding bone [5,6]. Furthermore, functionally graded implants have demonstrated several valuable advantages, such as enhancing bone cell development [7], reducing stress shielding [8], and providing the environments for osteoblast colonization and development [9]. These advancements highlight the promising potential of functionally graded materials in biomedical applications and beyond.

Drawing inspiration from naturally occurring topologies [10–14], TPMSs have been proposed and are widely acknowledged for their unique, topology-driven physical and mechanical characteristics. These structures possess a bi-continuous architecture, typically formed by alternating solid phase and space, with unit cells that repeat periodically in three dimensions and surfaces that possess zero-mean curvature throughout. This distinctive geometry grants TPMS structures exceptional properties such as smooth surface morphology, a favourable ratio of strength to weight, high energy absorption, and inherent structural interconnectivity [15–19]. Additionally, their special geometry supports efficient mechanical characterization modeling through artificial intelligence techniques [20,21] and enables fabrication via 3D additively printing methods [22,23]. Owing to these remarkable attributes, TPMS materials are used in diverse applications, including thermal management, impact mitigation, noise reduction, robotics, and biomedical implants [24].

The bone implants based on functionally graded TPMS architectures inherit the advantages of both TPMS structures and functionally graded materials. Wherein, the smooth surfaces and interconnected pores of TPMSs provide an ideal environment for bone development, meanwhile the nonlinearly graded geometry permits the precise stiffness adjustment to match that of natural bone. These favourable environments include a smooth surface that lessens impairment to adjacent bone cells, in the meantime enhancing cell growth, adhesion, and proliferation. Moreover, the interconnected pore network facilitates vascularization by enabling blood vessel infiltration and supporting bone cell integration within the implant, ensuring strong osseointegration. Thus, several research scholars have explored functionally graded TPMS-based bone implants, yielding promising outcomes in biomechanics and tissue regeneration. Indeed, Han et al. [25] presented bone scaffolds with a continuously graded porous diamond structure, demonstrating that adjusting the gradient index allows fine-tuning of the plastic strength and elastic modulus of titanium-based implants to match those of trabecular bone. They also highlighted that graded porosity can be adjusted to promote bone cell growth. In this regard, Viet et al. [26] studied the influence of the depth of bone ingrowth on the mechanical performance of FG bone implants considering numerous TPMS types. Their findings revealed a nonlinear increase in the plastic yield strength of the mixture of the bone and implant with increased depth of bone ingrowth. They also observed that implant stiffness could be tailored by modifying the gradation pattern. Similarly, Vijayavenkataraman et al. [6] investigated the mechanical properties of FG bone implants with TPMS structures exceeding 50% porosity, concluding that optimized gradation can mitigate stress shielding. Research on uniform TPMS-based bone implants has also provided valuable insights. Notably, gyroid-based implants closely mimic the human bone’s architecture [27], while the implants with TPMSs reduce shear and compressive stress on the articular cartilage in comparison with the solid implant [28]. In everyday activities, both the implant and bone experience multiaxial loading, so it is important to explore the multidirectional mechanical response of NFG bone implants prior to application [29,30]. Moreover, the behaviour and resilience of the implant under impact loads are also important topics to explore, which help protecting the patient from further injury and decreasing the requirement for invasive and costly revision surgeries [31–33].

Femoral implants with porous lattices have recently attracted significant attention from researchers. In this context, Chen et al. [34] introduced a 3D-printed implant using titanium material integrated with the Masquelet method for managing large femoral bone defects, reporting successful osseous integration at the implant–bone interface in 91% of patients. More recently, Jafari Chashmi et al. [35] conducted a numerical study on porous functionally graded material (FGM) implants and real femoral bone structures, demonstrating that optimizing the gradient index effectively reduces stress shielding between the implant and bone. Beyond functionally graded femoral implants, extensive research has explored the mechanical properties and novel applications of femoral implants with uniform lattice structures [36]. Meanwhile, porous architected materials have gained increasing attention for cranial bone implants and cranioplasty. Ayers et al. [37] investigated nitinol-based cranial implants, focusing on two key aspects: (1) bone ingrowth into the implant after six weeks and (2) the impact of pore size on early bone infiltration. Their findings indicated that pore size did not significantly affect bone ingrowth during the cartilaginous phase of healing, suggesting that within the commonly accepted porosity range (150–400 µm), bone integration near the nitinol implant interface remains consistent at six weeks. Shash et al. [38] conducted a numerical study to improve titanium-based cranioplasty performance by exploring alternative porous materials, including alumina, zirconia, hydroxyapatite, zirconia-reinforced PMMA, and PMMA. Under a 2000 N impact force, their results showed that alumina and zirconia implants more effectively reduced stress and strain on the skull and brain compared to titanium. Additionally, Liu et al. [39] introduced Medpor, a biocompatible porous implant, for reconstructing small to medium (<8 cm) convexity or cranial base defects resulting from skull base procedures. Medpor’s porous structure facilitates soft tissue and bone ingrowth, enhancing implant strength and reducing infection risk. The authors successfully used 611 Medpor implants in standard cranial and skull base surgeries, reporting excellent cosmetic outcomes with no implant-related complications.

While previous works have uncovered new characteristics and extensively expanded the biomedical research community’s understanding of regular FG implants, the impact of the topology and gradient index on the multidirectional mechanical response of TPMS-based SNFG femur and cranial bone implants under a multidirectional load remains insufficiently explored. Each component of an SNFG titanium bone implant with TPMSs offers distinct advantages. Titanium, famous for its exceptional biocompatibility, minimizes the chance of adverse reactions or rejection that makes it good compatible with the human bone and body. Moreover, the presence of voids in the SNFG TPMS topology stimulates the development of bone cell and support the exchange of needed oxygen, nutrients, waste, and fluids between the scaffold and neighbouring natural bone. Such exchange is important in keeping the biological process balances that are significant for the health, healing, and remodelling of the bone. Thus, this work presents a numerical investigation on the multidirectional mechanical response, such as in-plane and spatial anisotropy of elastic stiffness, the multiaxial yield surface, and impact wave characteristic of SNFG titanium femur and cranial bone implants using the TPMS topology, considering the impact of varying topology and gradient indices. Subsequently, conclusions are made to give an instruction for attaining ideal designs of TPMS-based SNFG titanium femur and cranial bone implants for biomedical applications. It is worthwhile noting that the mechanical properties of cranial and femur bones, such as stiffness and yield strength, vary among individuals depending on factors like age, genetics, and gender. Therefore, an optimization model should be developed to account for this variability, and this study can be a good foundation for building the optimization approach. Using advanced technologies, an artificial intelligence–based optimization approach is considered the most effective direction to pursue.

## Structural designs and numerical approaches

### Structural designs

**Fig 1:**
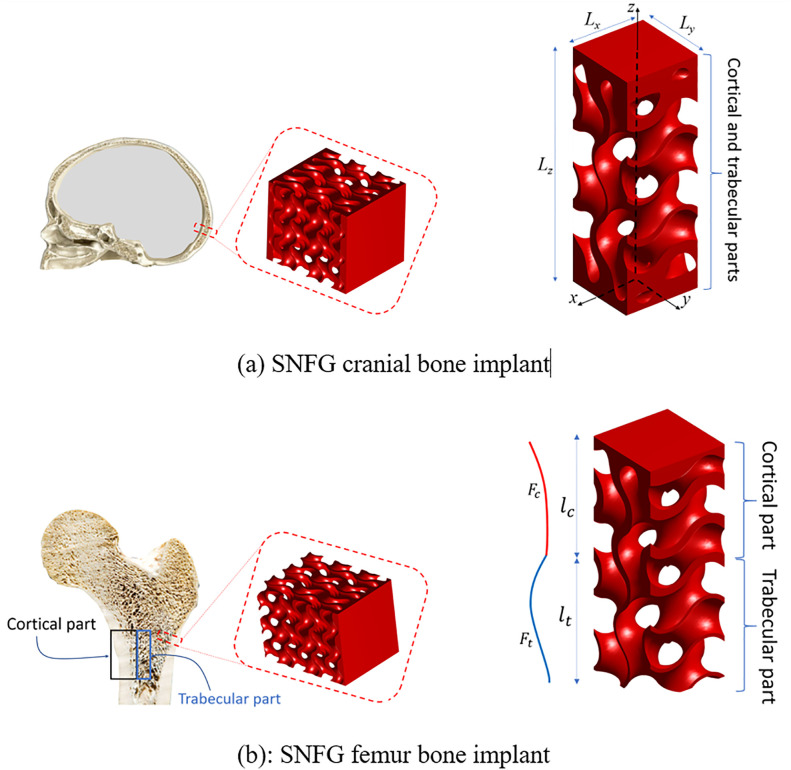
Demonstration of the topology of the unit cell of cranial (a) and femur (b) SNFG bone implants with gyroid, and the detailed structural design of each SNFG bone implant.

This study explores the multidirectional mechanical properties and feasibility of achieving an ideal SNFG titanium for cranial implants using TPMS structures. The implant designs mimic the natural architecture of femur and cranial bones, with representative unit cell topologies, including the gyroid structure, as illustrated in Fig 1.

**Fig 2:**
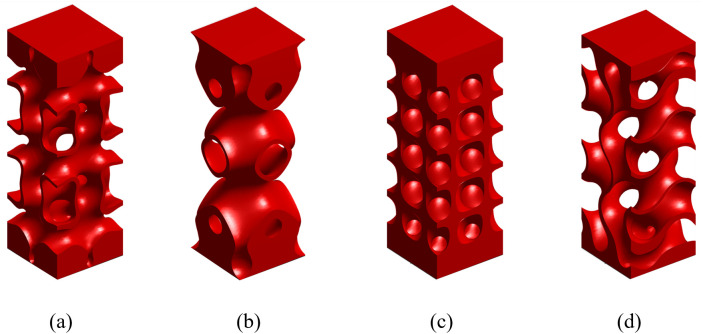
Schematic demonstration of different topologies of the unit cell of SNFG cranial bone implant with IWP(a), Primitive (b), Diamond(c), and Gyroid(d), all having ${𝐠}_{0}=4$.

All implant topologies are generated using a free solftware, MD-TPMS [40]. From the figure, each unit cell has a length $L_{z}$, width $L_{x}$, and depth $L_{y}$ in the Cartesian xyz−coordinate system (see Fig 1), and the grading direction is along the z-axis. It is noting that, due to the symmetric nature of TPMS structures, including the gyroid scaffold, grading the cell thickness along the x, y, or z direction results in similar mechanical behaviour. The unit cell of the femur bone implant consists of a cortical bone segment with a length of $l_{c}$ and a trabecular bone segment with a length of $l_{t}$, as shown in Fig 1(b). It is worth noting that lattice-based materials are more suitable for replicating trabecular bone, as trabecular bone is inherently a highly porous, lattice-like structure. However, it is important to note that natural cortical bone also contains a small degree of porosity, typically ranging from 5% to 10% [41,50]. Therefore, a lattice-based cortical bone implant that incorporates both dense and porous regions, such as the structural design presented in this work, and exhibits anisotropy and effective elastic stiffness similar in magnitude to that of natural cortical bone, can still be considered an appropriate and effective design. It is worth noting that upon obtaining an appropriate unit cell of the implant, it can be projected onto any implant architecture using nTopology software. The cortical and trabecular parts each have distinct gradient indices, $g_{c}$ and $g_{t}$, respectively, providing greater flexibility in designing an optimal bone implant. The SNFG cranial bone implant with TPMS structures is denser at the top and bottom, featuring a single gradient index, $g_{0}$, as illustrated in Fig 1(a). To determine the most suitable topology among commonly used TPMS architectures for bone implants, this study considers four most popular sheet-type TPMS structures, including IWP, primitive, diamond, and gyroid scaffolds, with their unit cell topologies presented in Fig 2.

where, IWP is an acronym of I-Wrapped Package, and is a type of TPMS structure characterized by its periodicity in three spatial dimensions and minimal surface area within a given boundary. Here, we define $F_{c}\;$and $F_{t}$ as the nonlinear functions governing the thickness variation along the z−direction in the cortical and trabecular regions of the femur implant, respectively, with their variations controlled by the gradient indices $g_{c}$ and $g_{t}$. Similarly, $F_{0}$ represents the mathematic function of nonlinear thickness distribution along the z−direction in the cranial bone implant, associated with the gradient index $g_{0}$. The mathematical formulations of these graded functions for each SNFG titanium bone implant in the xyz−coordinate system are presented as follows.

For cranial bone implant:


$$F_{0}=\left\{ \begin{array}{c} \left(t_{max}-t_{min}\right){\left[\left(z-L_{z}/2\right)/(L_{z}/2)\right]\;}^{g_{0}}+t_{min}\;with\;L_{z}/2<z<L_{z}\;\\ \left(t_{max}-t_{min}\right){\left[\left(-z+L_{z}/2\right)/(L_{z}/2)\right]\;}^{g_{0}}+t_{min}\;with\;0<z<L_{z}/2 \end{array}\right.\;$$ (1)

For femur bone implant:


$$\left\{ \begin{array}{c} F_{c}=\left(t_{maxc}-t_{minc}\right){\left[\left(z-L_{z}/2\right)/(L_{z}/2)\right]\;}^{g_{c}}+t_{minc}\;with\;L_{z}/2<z<L_{z}\;\\ F_{t}=\left(t_{maxt}-t_{mint}\right){\left[z/(L_{z}/2)\right]\;}^{g_{t}}+t_{mint}\;with\;0<z<L_{z}/2 \end{array}\right.\;$$ (2)

where terms $t_{max}$, and $t_{maxc}$ are the maximum thickness distribution of cranial and femur bone implant, respectively; $t_{min}$, and $t_{mint}$ are the minimum thickness distribution of cranial and femur bone implant, respectively. It is worth noting that various nonlinear functions can be used to define the thickness distribution in a graded structure, and the function used in this work is just one of many possible choices. However, based on our test and observation, the nonlinear functions presented in Eqs. (1) and (2) are good choices due to two key reasons. First, these functions offer flexibility, allowing us to nonlinearly adjust the thickness distribution by tuning the gradient index and the maximum and minimum thickness values. This enables us to replicate the architecture of natural cranial bone. For instance, the function allows the implant to be denser at both ends and more porous in the middle, closely resembling the natural structure of cranial bone. Second, the proposed functions facilitate smooth functional grading between adjacent sections of the implant. This smooth transition in thickness helps to reduce internal stress concentrations, which is essential for improving implant performance. The value of maximum and minimum thickness distribution used in this work is listed in Table 1.

**Table 1:** Cell thickness distribution at the top and end of the SNFG femur and cranial bone implants with different topologies.

	IWP	primitive	gyroid	diamond
$t_{max}=t_{maxc}$	5	3	1.5	1.1
$t_{min}=t_{mint}$	0.6	0.2	0.2	0.2
$t_{minc}=t_{maxt}$	1	0.5	0.5	0.5

**Fig 3:**
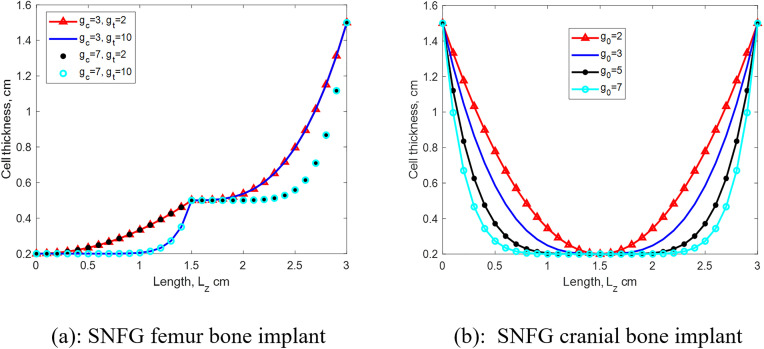
Presentation of nonlinear cell thickeness distribution along the graded direction of SNFG femur (a) and cranial (b) bone implants upon varying gradient indexes.

To better illustrate the distribution and the impact of the gradient index on cell thickness variation along the z-direction, examples of cell thickness distribution across the unit cell length for different gradient indices are presented in Fig 3.

### Numerical homogenization

**Fig 4:**
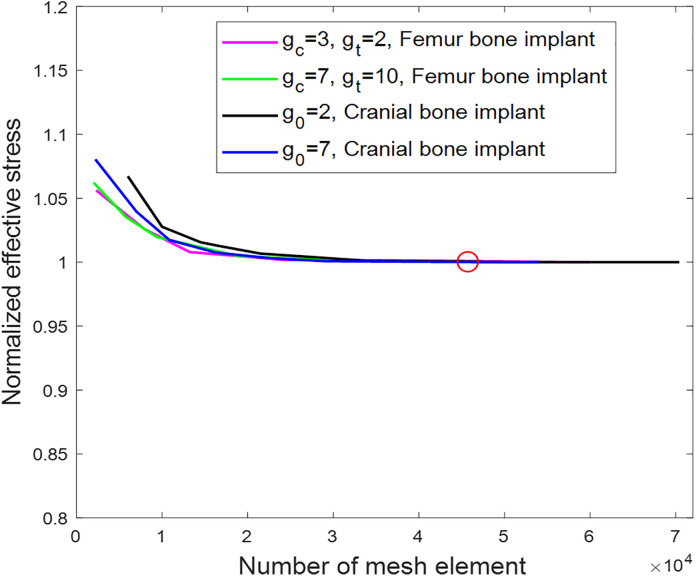
Convergence study of 3D FEM simulation in terms of the normalized effective stress versus the number of mesh element of femur bone implants with ${𝐠}_{c}=3$, ${𝐠}_{t}=2$ and ${𝐠}_{c}=7$, ${𝐠}_{t}=10$, and cranial bone implants with ${𝐠}_{0}=2$ and ${𝐠}_{0}=7$.

In this study, the SNFG implants are considered orthotropic materials due to the presence of porosity and the cell thickness’s nonlinear variation along the z-direction. In the numerical approach, the homogenization technique is employed to determine the effective mechanical properties, including effetcive strain and stress. The mathematical concept of the homogenization is expressed as:


$${\overset{―}{\sigma }}_{ij}=\frac{\text{1}}{V}\int_{V}^{\text{\textbackslash{} }}\sigma_{ij}dV=\frac{\text{1}}{qV}\{\sum_{m=\text{1}}^{m=q}\sigma_{m}\}V_{e}\text{, and }{\overset{―}{\varepsilon }}_{ij}=\frac{\text{1}}{V}\int_{V}^{\text{\textbackslash{} }}\varepsilon_{ij}dV=\frac{\text{1}}{qV}\{\sum_{m=\text{1}}^{m=q}\varepsilon_{m}\}V_{e}$$ (3)

where notations $\varepsilon_{ij}$ and $\sigma_{ij}\;$ are the effective strain and stress components in the elemental volume, dV, respectively; V is the unit cell volume; $V_{e}$ is the volume of an element; and q is the total number of nodes in an element. In this work, tetrahedral elements (C3D10) are used, which leads to *q = 4*. According to the equation, the homogenized value is evaluated as the average of the effective value multiplied by the element volume, divided by the total structural volume. The stress and strain components in the equation are obtained from a three-dimensional FEM simulation performed by the commercial software Abaqus that employs the tetrahedral elements to mesh the structures. The key mechanical properties computed and extracted in this study include the implant’s effective strain, stress, and elastic shear and tensile stiffnesses. From the achivement of these effective values, the anisotropy of phase wave propagation velocity and yield surface is subsequently computed. To reduce computational costs while accurately representing the mechanical behavior of an infinite implant structure, a unit cell approach is adopted in the 3D FE simulations. This is supported by periodic boundary conditions (PBCs), which ensure that the unit cell’s edges behave like a seamlessly connected to opposite edges in an infinite structure. The mathematical concept of PBCs is expressed as follows:


$$u_{i}\left(x_{i}+W_{i}\right)=u_{i}\left(x_{i}\right)+\varepsilon^{a}W_{i}\text{, }t_{i}\left(x_{i}+W_{i}\right)={-t}_{i}\left(x_{i}\right)\text{, \textbackslash{} }\forall x_{i}e_{i}\in \partial E\;$$ (4)

where $W_{i}$ is the unit cell’s edge length in the x− or y−directions; $x_{i}$ are the position vector’s components; $e_{i}$ is the unit vector in the x− or y−directions; $t_{i}$ is the surface traction normal to ∂E; $\varepsilon^{a}\;$is the applied strain; and ∂E is the cell border. In this work, the boundary conditions are applied to reflect the actual constraints of femur and cranial bones, where the outer surfaces perpendicular to the z-direction are left free. Specifically, under uniaxial loading along the x- or y-direction, the surfaces perpendicular to the z-direction are set free, while the other surfaces are subjected to periodic boundary conditions, as elaborately described in the literatures for uniaxial loading [42,43]. For other loading scenarios, such as pure shear and biaxial loading, the boundary conditions follow the methods described in detail in the literatures [42,43]. To determine the mesh element number at which the mechanical values of the SNFG implants converge under the same applied load, Fig 4 presents the normalized effective stress as a function of the number of mesh elements for femur bone implants with $g_{c}=3$, $g_{t}=2$ and $g_{c}=7$, $g_{t}=10$, and cranial bone implants with $g_{0}=2$ and $g_{0}=7$.

In the figure, normalized effective stress is used to eliminate the large value gap among the curves. It is calculated by dividing the resultant stress by the minimum resultant stress for each case under the same applied load in the elastic domain. As shown in the figure, all resultant stresses converge at a mesh element number of approximately 45,000, indicated by a red circle. Therefore, to ensure accuracy, an average of 45,000 tetrahedral elements (C3D10) per unit cell is used in the 3D FEM simulations. From the obtained effective values calculated by Eq. (3), the anisotropy of the effective stiffness in the orthotropic structure of NFG implant against varying orientations is evaluated [44].


$$\frac{1}{\overset{―}{E}\text{\textbackslash{} }}=S_{11}m_{1}^{4}+S_{22}m_{2}^{4}+S_{33}m_{3}^{4}+(S_{44}+2S_{23})m_{2}^{4}m_{3}^{4}+(S_{55}+2S_{13})m_{1}^{4}m_{3}^{4}+(S_{66}+2S_{12})m_{1}^{4}m_{2}^{4}$$ (5)

In Eq.(5), notations $m_{1}$, $m_{2}$, and $m_{3}$ are the cosines of $\overset{―}{E}\text{\textbackslash{} }$ to the x−axis, y−axis, and  z−axis, respectively; $\overset{―}{E}\text{\textbackslash{} }$ is the directional effective Young’s modulus; $S_{ij}$ is the component of the compliance matrix.

### Multiaxial yield surface and impact phase wave

Throughout daily activities, the femur and cranial bones, along with their implants, are exposed to dynamic loading conditions. These can include regular movements such as waking, running, and jumping, as well as more severe impacts from less frequent events like accidents or blasts. These activities generate long-wavelength and multidirectional stress waves passing through both the implants and the surrounding bone. Thus, studying the wave propagation property in SNFG femur and cranial implants helps understanding and achieving an ideal implant design that endures efficiently the adverse impact, and enhances the implant’s lifespan and durability.

**Fig 5:**
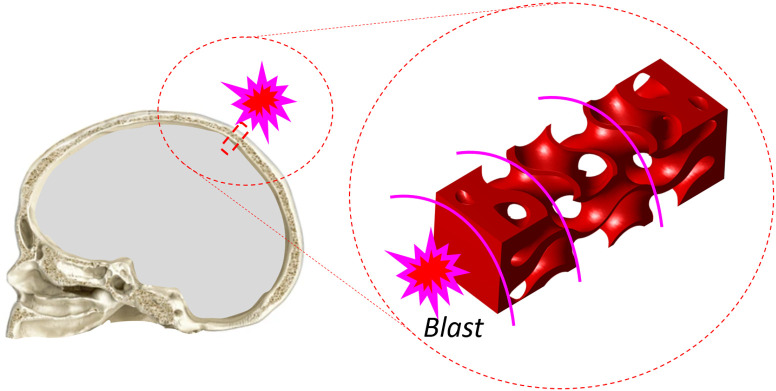
Schematic demonstration of an example of the longitudinal phase wave propagation in the cranial bone implant with gyroid subjected to an external blast.

In this work, the bone implant is considered to be subjected to an impact, resulting in the propagation of stress within the implant, as illustrated in Fig 5.

The motion of small elements triggered by the wave propagation can be described by a system of the mathematical equations using the Newton’s second rule given as:


$$\left\{ \begin{array}{c} \frac{\partial \sigma_{xx}}{\partial x}+\frac{\partial \sigma_{xz}}{\partial z}=\hat{\rho }\ddot{u}\\ \frac{\partial \sigma_{xz}}{\partial x}+\frac{\partial \sigma_{zz}}{\partial z}=\hat{\rho }\ddot{v} \end{array}\right.\;$$ (6)

In the equation, terms $\ddot{u}\;,$ $\ddot{v}$, and $\hat{\rho }$, stand for the x- and z- direction element accelerations with respect to time, and the mass density of implant, respectively. After several mathematical works, the shear $v_{s\theta }\;$ and directional longitudinal $v_{l\theta }$ stress wave velocities in SNFG femur and cranial bone implants considered as the orthotropic material can be expressed [45]:


$$\left\{ \begin{array}{c} v_{s\theta }=\sqrt{\frac{{-\chi }_{1}-\sqrt{\chi_{1}^{2}-4{\rho^{2}\chi }_{2}}}{2\rho^{2}}}\\ v_{l\theta }=\sqrt{\frac{{-\chi }_{1}+\sqrt{\chi_{1}^{2}-4{\rho^{2}\chi }_{2}}}{2\rho^{2}}} \end{array}\right.\;$$ (7)

In Eq. (7), the terms $\chi_{1}$ and $\chi_{2}$ are mathematically expressed as


$$\chi_{1}=-(C_{11}{cos}^{2}\theta +C_{44}{sin}^{2}\theta +C_{33}{cos}^{2}\theta +C_{22}{sin}^{2}\theta )\rho$$ (8)

and


$${\;\chi }_{2}=\left(C_{11}{cos}^{2}\theta +C_{44}{sin}^{2}\theta \right)\left(C_{33}{cos}^{2}\theta +C_{22}{sin}^{2}\theta \right)-(C_{12}cos\theta sin\theta +C_{44}cos\theta sin\theta )(C_{33}cos\theta sin\theta +C_{21}cos\theta sin\theta ).$$ (9)

Where notations $C_{ij}$ are the in-plane effective stiffness component. It is noted that the soundness of Eq. (7) was numerically and experimentally confirmed [45].

In reality, the implant can be exerted by multidirectional loading conditions, causing complex multidirectional stress states. As a result, the in-plane anisotropy of multiaxial yield surfaces of the considered femur and cranial bone implants are very important to be explored, showing multidirectional response of the implant subjected to these complex multidirectional stress conditions, which helps anticipate the failure and offer optimal designs for increasing the implant’s lifespan. In this work, the bidirectional plastic yield strength data of a SNFG titanium femur and cranial implants are calculated by the presented numerical approach. Wherein the material properties of titanium, including its experimental axial stress-strain response in the elastoplastic domain, are taken from work [46]. Wherein, the Young’s modulus and Poisson’s ratio of titanium are taken as 104 GPa and 0.3, respectively. Direct implementation of the numerical model to attain the yield surface data requires a huge cost. To address this, the yield surface of the implant is established by using the extended anisotropic Hill’s constitutive model [47], which requires only seven coefficients equivalent to seven numerical data to form the surface. The extended Hill’s criterion is expressed as


$$A{\left(\sigma_{xx}-\sigma_{yy}\right)}^{2}+B{\left(\sigma_{yy}-\sigma_{zz}\right)}^{2}+C{\left(\sigma_{xx}-\sigma_{zz}\right)}^{2}+{D\sigma }_{xy}^{2}+{E\sigma }_{yz}^{2}+{F\sigma }_{xz}^{2}+{G\sigma }_{H}^{2}=1$$ (10)

Where notations A, B, C, D, E,F, G are constants expressing the degree of anisotropy of the considered implant; $\sigma_{H}=(\sigma_{xx}+\sigma_{xx}+\sigma_{xx})/3$ is the hydrostatic stress; and $\sigma_{ij}$ and $\sigma_{ii}$ are shear and axial stresses.

## Experimental validation

**Fig 6:**
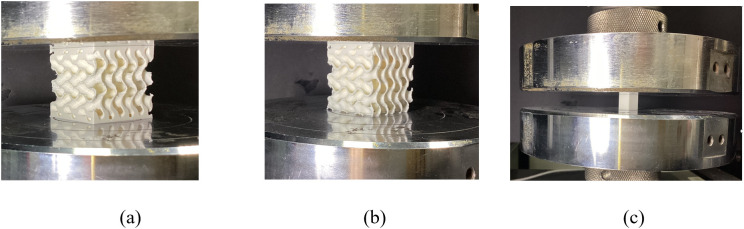
Schemiatic demonstration of the compressive test of samples. (a): SNFG cranial bone implant; (b): SNFG femur bone implant; (c): Dense white PLA **cube.**

In the experimental work, we first used MD-TPMS software to create the cubic SNFG femur and cranial implant samples, having a dimension of 3 cm × 3 cm × 3 cm in STL format. The topologies in STL format were then moved to an Ultimaker S5 Pro 3D printer for printing. In all tests, all samples with gyroid structures were subjected to compressive loading using a 50 kN load cell on an INSTRON testing machine at room temperature. The experimental setup and representative samples are shown in Fig 6.

The base material used for printing the samples is PLA. Its elastic stiffness, both along and perpendicular to the printing direction, is measured through separate compressive tests on 1 cm x 1 cm x 1 cm cubes, as depicted in Fig 6(c). In the tests, the white PLA dense cubes are compressively loaded along and perpendicular to the printing direction. The displacement rate of 0.05 mm/minute is set for all tests. In loading state, compressive force-displacement data curves are precisely documented, which are used to calculated the effective stress by dividing the applied force by the cross-sectional area of the sample. Similarly, we measured the strain by dividing the applied displacement by the length of sample. It is known that under the same relative density and topology, the Young’s modulus of SNFG bone implants with titanium can be linearly related from that with PLA, given by a formulation, $E_{ti}=E_{pla}.E_{ti}^{0}/E_{pla}^{0}$ [48]. Where notations $E_{pla}$, $E_{pla}^{0}$, and $E_{ti}^{0}$, represent the elastic stiffness of implant with PLA, implant with titanium, dense white PLA, and dense titanium, respectively. As a result, samples fabricated using PLA can be employed to verify the soundness of predicting the mechanical properties of implant with titanium using numerical model [49]. During the post-processing of the experimental tests, the Young’s modulus of white PLA is measured under compressive loads applied both along and perpendicular to the printing direction. The measured values are 2.53 GPa and 2.23 GPa, respectively. Additionally, the Poisson’s ratio of PLA is assumed to be 0.35 [49].

## Result and discussions

**Fig 7:**
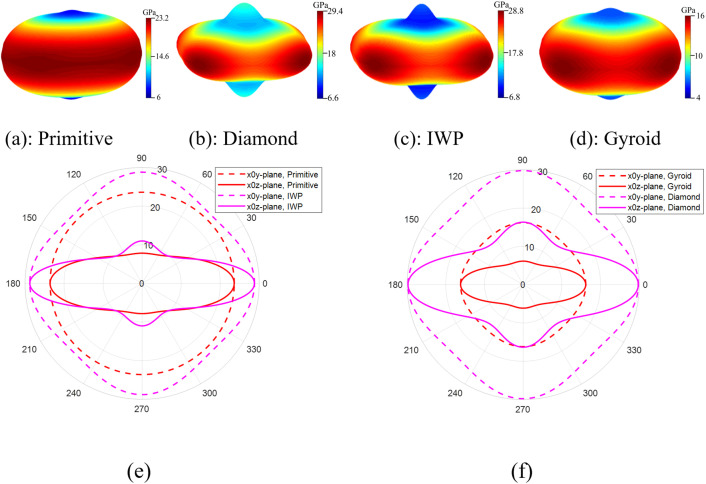
Spatial anisotropy (a)-(d) and, on directional anisotropy (e) and (f) of SNFG titanium cranial bone implants with primitive, diamond, IWP, and gyroid topologies, all with ${𝐠}_{0}\;=4$, against varying orientations. The vertical axis represents either 𝐳 **or** 𝐲−**direction, and transverse axis represents the** 𝐱−**direction.**

Here, the percent difference is calculated by dividing the absolute difference between two values by the larger value and multiplying the result by 100. Additionally, experimental work is conducted only using PLA as the base material, while other presented results were obtained using titanium as the base material. To assess the impact of TPMS topology on the multidirectional mechanical response of SNFG titanium bone implants under varying loading conditions, Fig 7 presents the in-plane and three-dimensional anisotropy of the effective stiffness in terms of Young’s modulus for SNFG titanium cranial bone implants based on primitive, diamond, IWP, and gyroid, all with $g_{0}=4$, against varying orientations.

The figure illustrates that SNFG cranial bone implants with primitive and gyroid structures exhibit more uniform mechanical responses across different orientations compared to other designs, as indicated by their lower anisotropy. However, the gyroid implant has a lower Young’s modulus than the primitive implant, offering a broader range of elastic stiffness. This characteristic allows for better tuning of the implant’s stiffness to match that of a biological bone, particularly in regions dominated by very soft trabecular bone. It is also worth noting that, due to natural optimization, biological bone exhibits less pronounced anisotropy, as shown in Fig 10(d). For brevity, this study focuses on the SNFG bone implant with a gyroid topology to investigate the conditions necessary for achieving an optimal SNFG titanium femur and cranial bone implant. The reduced anisotropy associated with the gyroid structure offers several advantages, including improved fatigue resistance, lower internal stress variations, enhanced processability, and other beneficial mechanical properties.

**Fig 8:**
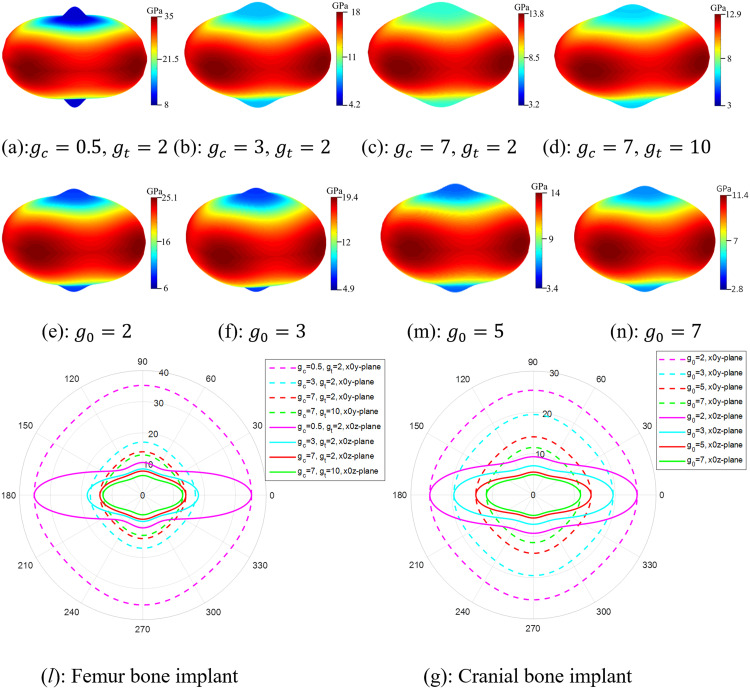
Effect of gradient index on the multidirectional mechanical response in terms of spatial anisotropy of effective Young’s modulus (radius in GPa) of femur (a)-(d) and cranial (e)-(n) bone implants, and in aspects of in-plane anisotropy of femur (*l*) and cranial (g) bone implants. **The vertical axis represents either the** 𝐳 **or** 𝐲−**direction, and transverse axis represents the** 𝐱−**direction.**

**Fig 9:**
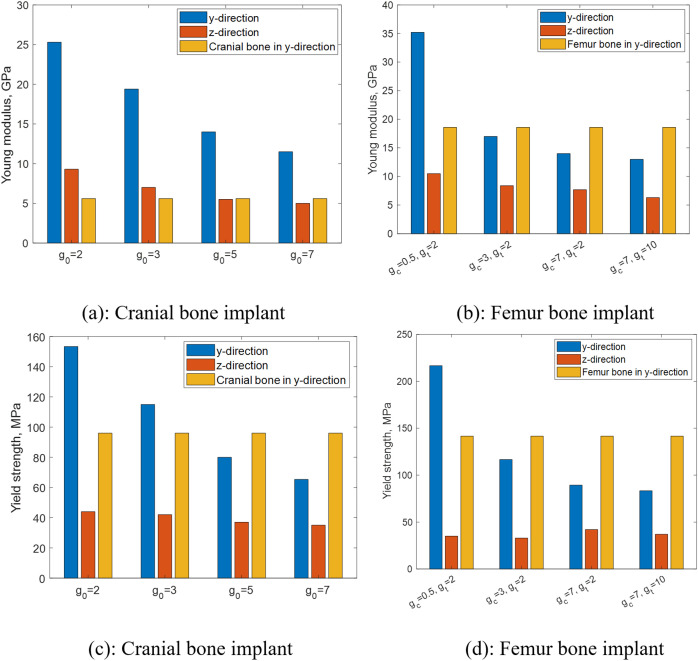
Effective axial Young’s modulus and yield strength of SNFG bone implants under varying gradient indices and a comparison to an average value of respective bones. **(a) and** (c): Cranial bone implant; (b) and (d): **Femur bone implant.**

Fig 8 illustrates the effect of the gradient index on the overall multidirectional mechanical property of SNFG titanium femur and cranial bone implants, in terms of the 2D and 3D anisotropy of the effective Young’s modulus under multidirectional loading.

Based on the figure, increasing the gradient index leads to a drop of the elastic stiffness in a nonlinear pattern, along with more uniform multidirectional mechanical responses of two SNFG titanium femur and cranial bone implants, reflected via a rounder profile of the anisotropy against the varying orientations. In particular, when the gradient index of SNFG titanium cranial bone implant increases from 2 to 7, the y-direction Young’s modulus drops from 25.3 GPa down to 11.5 GPa, respectively. Similar to the femur bone implant, the Young’s modulus in the y-direction drops from 35.2 GPa down to 14 GPa as the gradient index increases from $g_{c}=0.5$ to $g_{c}=7$, both associated with $g_{t}=2$, respectively. The observed differences in anisotropic profiles for each implant type, associated with different gradient indices, are due to the higher gradient index causing the solid thickness to distribute more uniformly across the implant, resulting in a structure that approaches uniformity. More importantly, the anisotropy in the y0z and x0z-planes is significantly more pronounced than in the x0 y-plane, reflected via the more distorted shape of the profile in the figure. During daily activities such as running, walking, and even accidents, bones and implants are primarily subjected to the multidirectional loading [50]. Therefore, greater attention should be given to the design of implants subjected to multidirectional loads in the x0 z and y0 z-planes rather than in the x0 y-plane to improve their performance in service.

Fig 9 illustrates the effective axial Young’s modulus and yield strength of SNFG bone implants under varying gradient indices and a comparison to an average value of respective bones.

It is worthwhile noting from the figure that the mechanical property comparison in the graded or z-direction is not reported, because we could only find the experimental value of the Young’s modulus of cranial bone in the z-direction, as reported in [51]. However, the corresponding experimental value for the femur bone in the z-direction could not be found. To maintain consistency between the comparison figures for the femur and cranial bone implants, our comparison in aspects of the effective Young’s modulus and yield strength is limited to that in the y-direction. In the figure, the average values of Young’s modulus and yield strength for the femur bone are 18.6 GPa [52] and 141.6 MPa obtained from the literature [53], while those for the cranial bone are 5.2 GPa, and 96 MPa from the literature [51]. The figure highlights an important finding that adjusting the gradient index allows for tuning the elastic stiffness of SNFG femur and cranial bone implants to closely match that of their respective natural bones while maintaining a yield strength higher than that of the bones. It is worth noting that the elastic stiffness of an ideal bone implant is commonly from 1 to 5 times greater than that of the natural bone being replaced [54]. Indeed, for the titanium cranial bone implant, when $g_{0}$ varies between 3 and 5, and for the titanium femur bone implant, when $g_{c}$ ranges from 0.5 to 3, the implant’s Young’s modulus closely approaches that of the respective natural bone, while the yield strength exceeds that of the host bone. These findings provide a crucial basis for developing an optimization approach to determine the ideal SNFG titanium cranial and femur bone implants by adjusting the gradient index to reasonably match the given elastic stiffness and the higher yield strength compared to those of the bones to minimizing stress shielding and preventing permanent plastic deformation.

**Fig 10:**
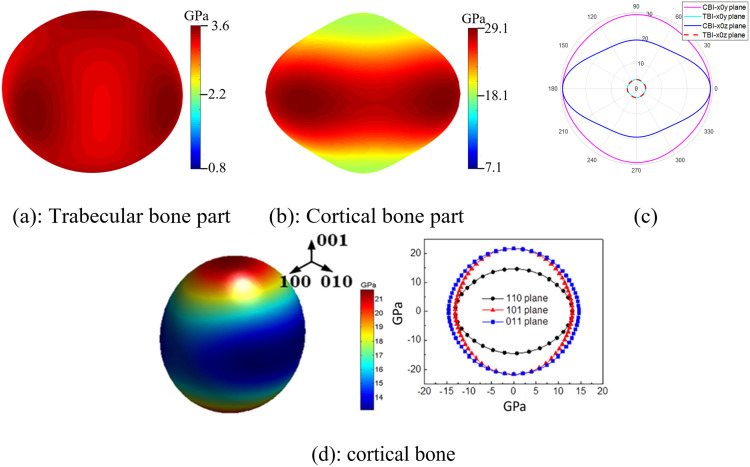
Spatial (a) and (b), and directional (c) anisotropy of effective Young’s modulus (radius in GPa) of trabecular and cortical bone implant parts with gyroid, and cortical bone(d) [51,52]. The decomposition is performed for SNFG femur bone implant with gc = 3 and gt = 10.

Fig 10 shows in-plane and spatial anisotropy of effective Young’s modulus of trabecular and cortical bone implant parts with gyroid, and cortical bone, with the decomposition performed for SNFG femur bone implant with $g_{c}=3$ and $g_{t}=10$.

It is noting that porous materials, including most TPMS architectures, often exhibit extreme anisotropy in elastic stiffness, particularly at low relative densities. This characteristic makes them less suitable for bone implants, which naturally possess low anisotropy, as indicated by the nearly round and smooth profile in Fig 10(d). Interestingly, Fig 10 illustrates that the directional and spatial anisotropy profiles of the cortical bone implant (CBI) closely resemble those of natural cortical bone, both displaying a near-round and smooth shape. This further supports the implant’s suitability for femur and cranial bone applications, where cortical bone is the predominant component. Moreover, the ratio of the highest to the lowest elastic stiffness values is 1.65 for natural cortical bone and 1.52 for the cortical region of the femur bone implant, demonstrating a close rate. Additionally, the results reveal that anisotropy of the trabecular implant (TBI) part is less pronounced than that of the cortical part, as indicated by its less distorted shape, further reinforcing the suitability of the SNFG implant with a gyroid as an ideal candidate for bone implants.

A comparison of the anisotropic ratio of the mean Young’s modulus in the longitudinal direction to that in the transverse direction for the cortical region of the proposed femoral bone implant (gyroid structure with $g_{c}=3$ and $g_{t}=10$ and human femoral cortical bone, based on data from three references [55–57], is presented in Table 2.

**Table 2:** Comparison of the ratio of Young’s modulus in the longitudinal direction to that in the transverse direction for the cortical region of the proposed femoral bone implant and human femoral cortical bone.

	Young’s modulus in Longitudinal direction (GPa)	Young’s modulus in transverse direction (GPa)	Ratio
Cortical part of gyroid implant	28.9	19	~1.52
Reilly and Burstein [55]	17	11.5	~1.47
Mirzaali et al. [56]	19	13	~1.46
Dong et al. [57]	19.1	6.49	~2.9

The table shows a comparable anisotropic ratio between the human femoral cortical bone and the proposed femoral bone implant with a gyroid scaffold having $g_{c}=3$ and $g_{t}=10$. Specifically, the ratio for the presented implant is approximately 0.152, while the corresponding ratios reported for natural human femoral cortical bone are 1.47 [55], 1.46 [56], and 2.9 [57], showing a close ratio among works. Interestingly, this ratio of the presented implant can be adjusted to more closely match that of natural bone by modifying the gradient indices $g_{c}$ and $g_{t}$, as illustrated by their influence in Fig 10. This finding highlights the strong potential of the proposed implant design for personalized applications in human femoral bone replacement.

**Fig 11:**
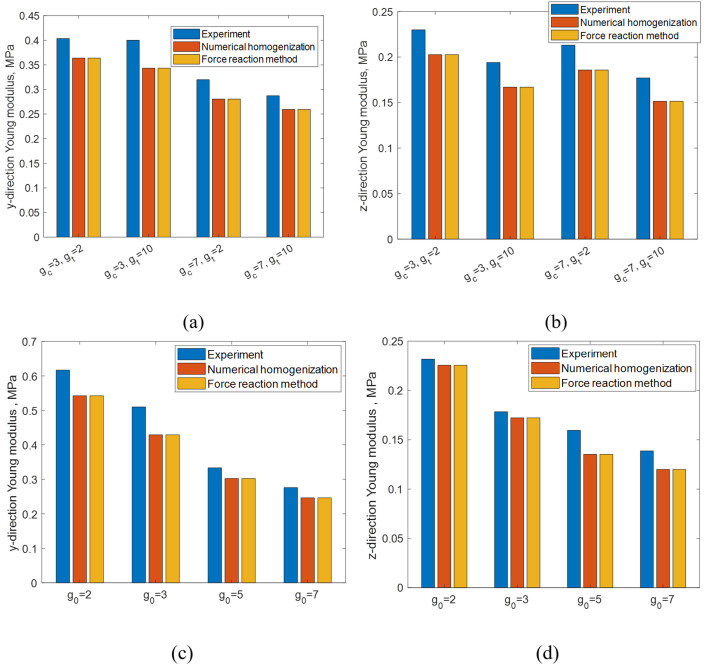
A comparative analysis of experimental results, the reaction force method, and adopted numerical homogenization in terms of effective compressive Young’s modulus of implants in parallel and perpendicular to graded direction upon varying gradient indices. **(a)** and (b): SNFG femur bone implant; (c) and (d): SNFG **cranial bone implant.**

Fig 11 presents a comparative analysis of experimental results, the force reaction method, and the presented numerical approach in evaluating the effective compressive Young’s modulus of implants, both parallel and perpendicular to the graded direction, across different gradient indices.

The figure shows an agreement among three prediction methods in aspects of the elastic stiffness, regardless of the different loading directions, having a maximum percent difference of 15.6% for the case of $g_{0}=3$ of SNFG cranial bone implant in the y-direction between experiment and numerical model. Noticeably, the force reaction method, which is built-in function in Abaqus, shows an almost identical results as that of numerical homogenization, with maximum percent difference being 0.1%. Both abovementioned comparison results highlight the numerical approach adopted in this work as an accurate, low-cost and reliable technique, for exploring mechanical properties of SNFG titanium femur and cranial bone implants with TPMSs. The discrepancy between results obtained from experiment and numerical model may be due to several factors, including printing defects, slippage during experimental tests, and the limited number of cells in the experiment [49], while such adverse conditions do not occur in numerical homogenization.

**Fig 12:**
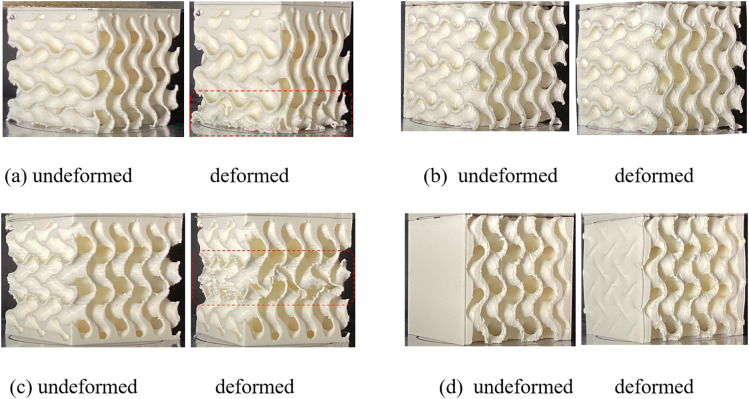
Undeformed and deformed samples under an applied compressive strain of 10% in the loading direction along and perpendicular to the graded direction. **(a) and** (b): **SNFG femur bone implant samples with gyroid and** ${𝐠}_{c}=7$, ${𝐠}_{t}=2$ **under the loading direction parallel and perpendicular to the graded direction, respectively; (c) and (d): SNFG cranial bone implant samples with gyroid having** ${𝐠}_{0}=3$ **under the loading direction parallel and perpendicular to the graded direction, respectively.**

Fig 12 displays some examples of undeformed and deformed samples under compression in the loading direction along and perpendicular to the graded direction of SNFG femur bone implant samples with $g_{c}=7$, $g_{t}=2$, and SNFG cranial bone implant samples having $g_{0}=3$.

The figure illustrates that SNFG implant samples exhibit sensitivity to loading along the graded direction, with the thinnest section experiencing significant deformation, as highlighted by the red squares in Figs 12(a) and 12(c). In contrast, both types of implants undergo uniform deformation when loaded perpendicular to the graded direction, which brings a benefit since, in real-world applications, bone implant primarily experience forces from the boundary between it and the neighbouring bones, interacting surrounding the x or y-directions [58,44].

**Fig 13:**
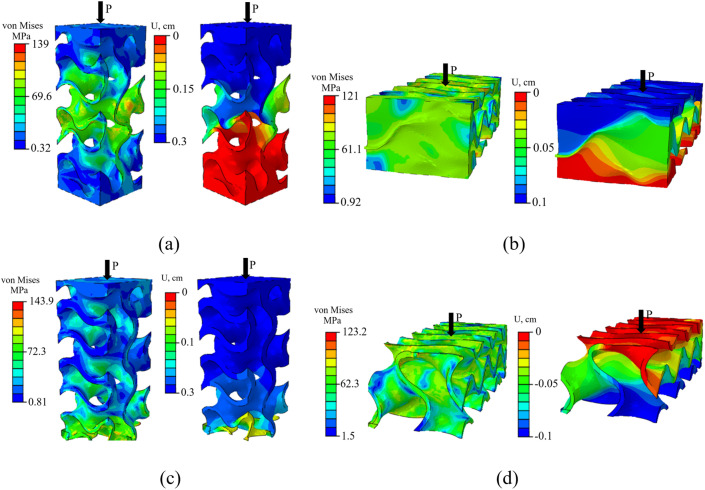
Contour plots of von Mises stress distribution and deformation in PLA-based SNFG cranial bone implant (${𝐠}_{0}$ = 3) and femur bone implant (${𝐠}_{c}$ = 7, ${𝐠}_{t}$ = 2) under compressive loading in the z-direction (a, c) and y-direction (b, d) at an applied compressive strain of 10%.

To deeply view the mechanical behaviour of samples under compressive loading, the contour plots of the von Mises stress distribution and deformation of unit cells of PLA-based SNFG cranial and femur bone implants under loading in the z-direction and the y-direction are shown in Fig 13.

It is worth noting that contour plots of deformation and von Mises stress at an applied strain of 10% are presented in Fig 12 to help visualize the stress distribution and deformation behaviour of the implant samples during the experimental tests conducted at the same strain level illustrated in Fig 12. The figure exhibits an agreement with results presented in Fig 12 that the significant stress concentration and deformation are located at the thinnest region of two types of the implant. Additionally, stress distribution and deformation are much more uniform under the y-direction load than that under the graded direction load. It is also noting that the mechanical behaviour of PLA cannot be directly related to that of titanium in the plastic domain, particularly in terms of stress distribution and deformation behaviour, due to the significant differences in their material properties. Thus, the stress distribution and deformation behaviour of the PLA implant may be different to that of the titanium implant in the plastic domain.

**Fig 14:**
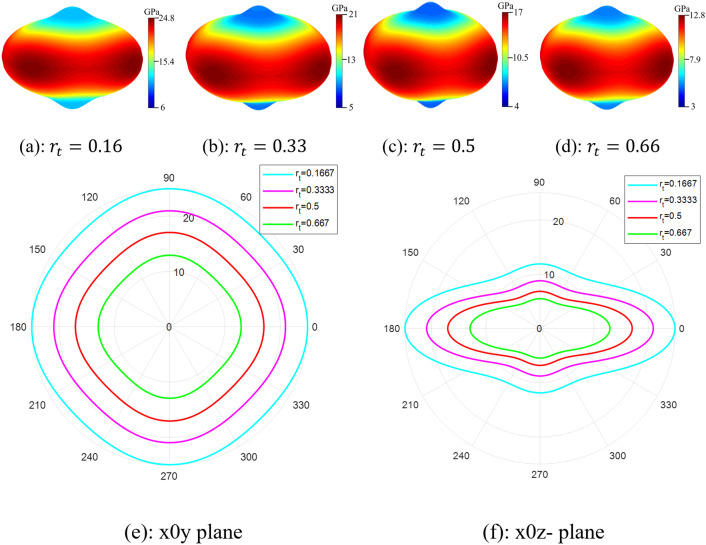
Effect of the length ratio of trabecular part on spatial (a)-(d), and directional (e) and (f) anisotropy of tensile stiffness (radius in GPa) of SNFG titanium femur bone implant based on gyroid.

Fig 14 illustrates the effect of the trabecular part’s length ratio on the spatial (a–d) and directional (e, f) anisotropy of the effective Young’s modulus (radius in GPa) in the SNFG titanium femur bone implant with a gyroid structure.

In the figure, the length ratio of the trabecular part, denoted as $r_{t}$, is the proportion of the trabecular part’s length relative to the total implant length, $L_{z}$. The figure highlights a crucial finding that the implant’s multidirectional mechanical response remains unaffected by variations in the trabecular length ratio within the considered range of the ratio. This is evident from the unchanged anisotropic profile of the elastic stiffness. Furthermore, the figure reveals that decreasing the ratio nonlinearly increases the elastic stiffness of the bone implant. Specifically, when $r_{t}$ varies from 0.1667 to 0.667, the implant’s stiffness changes from 25 GPa to 12.7 GPa, respectively. Interestingly, based on the figure, the y-direction Young’s modulus of femur bone, approximately 18.6 GPa, falls within the range of the effective Young’s modulus of the femur bone implant having the ratio ranging from $r_{t}=0.333$ to $r_{t}$=0667. Thus, adjusting the trabecular length ratio enables fine-tuning of the implant’s elastic stiffness to closely match that of the femur bone.

**Fig 15:**
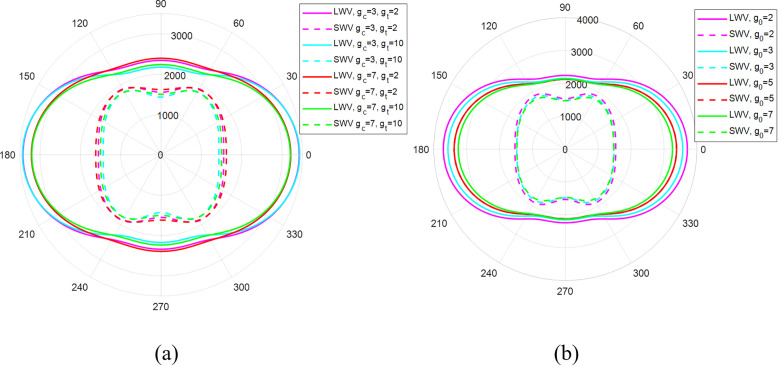
Effect of the gradient index on the in-plane anisotropy of shear and longitudinal impact phase wave velocities (radius in m/s) propagating in the z0x plane of SNFG titanium femur (a) and cranial (b) bone implants with gyroid, subjected to an impact load. The vertical axis represents for the z-direction, and transverse axis represents for either the x or y-direction.

The effect of the gradient index on the in-plane anisotropy of shear and longitudinal impact phase wave velocities passing through the z0x plane of SNFG titanium femur and cranial bone implants with gyroid, subjected to an impact load is shown in Fig 15.

The figure illustrates an almost unchanged anisotropic profile of wave velocity across different propagation directions, as evidenced by the similar shapes of the 2D wave profiles. Additionally, decreasing the gradient index increases the velocity of both longitudinal and shear waves traveling via SNFG femur and cranial bone implants. For example, as the gradient index increases from 2 to 7, the y-direction longitudinal wave velocity of the SNFG cranial bone implant with a gyroid structure drops from 3709 m/s to 3257 m/s. The faster longitudinal and shear wave velocities with a smaller gradient index result in greater stress concentration level in a smaller period, which can accelerate damage and reduce the implant’s lifetime. Thus, choosing an appropriate gradient index that reduces wave propagation velocity is crucial for enhancing the longevity and performance of considered SNFG bone implants. Another notable behaviour is that the graded-direction longitudinal wave is slower than in other axial directions, irrespective of the gradient index. This suggests that the designed implant endures better the impacts from daily activities such as jumping, running, and accidents triggering forces in the graded direction. Additionally, the figure reveals that the anisotropic profile of the shear wave is the inverse of the profile of the longitudinal one. Specifically, while the fastest longitudinal wave propagates in the y- and x-directions, these directions exhibit the slowest shear wave velocities.

**Fig 16:**
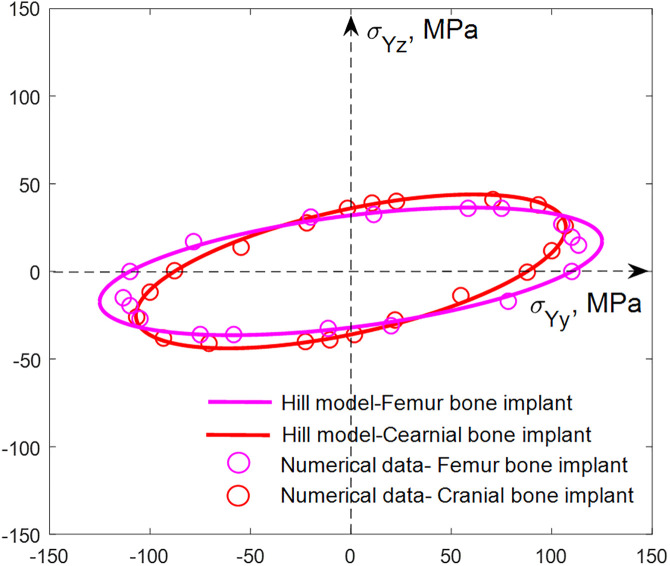
Demonstration of the in-plane anisotropy of multiaxial yield surfaces against varying orientations of SNFG femur bone implant with gyroid,${𝐠}_{c}=3$ and ${𝐠}_{t}=10$, and cranial bone implants with gyroid and ${𝐠}_{0}=4$ obtained from numerical model and fitted by the extended Hill’s constitutive law.

The anisotropy of multiaxial yield surfaces against varying orientations in the y0z plane of SNFG femur bone implant with gyroid, $g_{c}=3$ and $g_{t}=10$, and cranial bone implants with gyroid and $g_{0}=4$ obtained from numerical model and fitted by the modified Hill’s model is displayed in Fig 16.

Terms $\sigma_{Yz}$ and $\sigma_{Yy}$ in the figure are the z- and y-direction yield strengths, respectively. One can be seen from the figure that the in-plane anisotropy of the multiaxial yield strength of both femur and cranial bone implants with TPMSs is extreme, displaying via the yield strength data laying on a narrow elliptical shape aligning in a diagonal direction and possessing the weakest yield strength in the z or graded direction in comparison with other axial directions. In particular, the z and y direction yield strengths of cranial bone implant are 36 MPa and 87.8 MPa, respectively, demonstrating a significant discrepancy between axial yield strengths. This observed characteristic contrasts with that of conventional uniform porous architected materials [49], where the yield strengths in all axial directions are equal. Additionally, Hill’s model closely aligns with the numerical data, regardless of implant type or topology, demonstrating its effectiveness as a reliable and cost-efficient method for forming the multidirectional yield surface of the considered TPMS-based implants. Determining the multiaxial yield surface of these bone implants under multidirectional loading conditions is of paramount importance. It establishes a critical boundary that predicts when the implant will yield under complex loading scenarios, rather than relying solely on simple uniaxial stress-strain data.

## Conclusions

This study investigates the multidirectional mechanical properties of novel SNFG titanium femur and cranial bone implants with TPMS structures using a numerical approach. The numerical model is validated against experimental results and the force reaction approach in aspects of effective Young’s modulus, demonstrating good agreement. The maximum percentage difference, 15.6%, is observed for $g_{0}\;=3$ in the SNFG cranial bone implant along the y-direction when comparing experimental and numerical results. Furthermore, the numerical model predicts effective mechanical properties that closely match those obtained from the force reaction method using 3D FEM. These findings conclude that:

(i) Based on the multidirectional mechanical response, the gyroid topology emerges as the most suitable candidate for manufacturing SNFG bone implants. Compared to other topologies, including IWP, primitive, and diamond, it exhibits the closest match to the mechanical properties of biological bone.
(ii) The elastic stiffness of the proposed bone implants can be adjusted to closely match that of natural bone while maintaining a yield strength higher than that of bone. Similarly, stiffness tunability can also be achieved by modifying the length ratio of the trabecular region. Both approaches offer a flexible and straightforward method for optimizing the implant’s elastic stiffness in order to obtain ideal femur and cranial bone implants in the practical application.
(iii) This ratio of Young’s modulus in longitudinal direction to that in transverse direction of the cortical part of the implant can be adjusted to more closely match that of natural bone by modifying the gradient indices $g_{c}$ and $g_{t}$,. This finding suggests the strong potential of the proposed implant design for applications in human femoral bone replacement.
(iii) Increasing the gradient index drops shear and longitudinal wave velocities but has minimal impact on the anisotropic shape of the velocity distribution. Therefore, selecting the highest possible gradient index to minimize wave propagation velocity is essential for enhancing the longevity and performance of the implants.
(iv) The multiaxial yield strength anisotropy of both femur and cranial bone implants with TPMS structures is extreme and exhibits a distinct profile compared to uniform TPMS architectures. The multiaxial yield surfaces of these implants are well captured by the extended Hill’s criterion, highlighting its effectiveness as a reliable and cost-efficient method for constructing the critical yield surface of SNFG titanium bone implants with TPMS structures, which helps avoiding the permanent plastic deformation during service.

## Supporting information

S1 File MATLAB code for Homogenization technique. (PDF)
