# Supplementary material for: Tuneable multidirectional mechanical attributes of novel sectionally nonlinearly functionally graded femur and cranial bone implants with triply periodic minimal surfaces
Source: PLoS One. 2025 Sep 9;20(9):e0332104. doi: 10.1371/journal.pone.0332104 (PMC12419660; doi:10.1371/journal.pone.0332104)
Supplement: S1 File — (PDF) [file pone.0332104.s001.pdf]

```
clear all

close all

%%%%%%%% MATLAB code for Homogenization technique of Tetahedral element

v=[ ];% element volume

a=[ ];% either stress or strain data

% body code

bs=a(:,3); % extract stress column 3 and all raws

bz=a(:,4); % extract strain column 4 and all raws

ev=v(:,2); % extract volume of elements from colunm 2

ss=size(a);

sss=ss(1,1)/4; %tetrahedral element

n=sss*4; % total number of number (nodes)

ne=sss-1; % total number of elemnt from 0 and 1 ... noted that ne=1 mean 2 because it is 0 and 1.

for i=1:4:n

   cs(i,1)=bs(i,1)+bs(i+1,1)+bs(i+2,1)+bs(i+3,1); % turn all stresses in an elemnt into 1

   cz(i,1)=bz(i,1)+bz(i+1,1)+bz(i+2,1)+bz(i+3,1); % turn all strains in an elemnt into 1

end

for i=0:ne

ds(i+1,:)=cs(4*i+1,1)/4;  % avaeraging the stress and turn it into smaller size matrix for timing with
volume.
```

```
dz(i+1,:)=cz(4*i+1,1)/4; % avaeraging the strain and turn it into smaller size matrix for timing with volume.

end


sum_s=sum(ds.*ev);     % sume of stress-element volume product

sum_z=sum(dz.*ev);     % sume of strain-element volume product


% end of body code

 Lz=3;% length mm

LY=1;%%% width mm

LX=1;% height  mm


evol=sum(ev); % total volume of solid part

Vol=LZ*LY*LX; % total volume of structure


 %%%%% Effective values

s1=sum_s/Vol; % effective stress

z=sum_z/Vol; % effective strain
```
